# Gene expression patterns in heterozygous *Plk4 *murine embryonic fibroblasts

**DOI:** 10.1186/1471-2164-10-319

**Published:** 2009-07-16

**Authors:** Alan Morettin, Alejandra Ward, Jordan Nantais, John W Hudson

**Affiliations:** 1 Department of Biological Sciences, University of Windsor, Windsor, Ontario, Canada

## Abstract

**Background:**

The polo-like kinases (Plks) are a group of serine/threonine kinases which have roles in many aspects of cellular function including the regulation of mitotic activity and cellular stress responses. This study focuses on Plk4, the most divergent member of the Plk family, which is necessary for proper cellular proliferation. More specifically, alterations in Plk4 levels cause significantly adverse mitotic defects including abnormal centrosome duplication and aberrant mitotic spindle formation. We sought to clarify the effect of reduced Plk4 levels on the cell by examining transcript profiles of *Plk4 *wild-type and heterozygous mouse embryonic fibroblasts (MEFs). Subsequently, the levels of several key proteins involved in the DNA damage response were examined.

**Results:**

143 genes were found to be significantly up-regulated in the heterozygous MEFs compared to their wild-type counterparts, while conversely, 9 genes were down-regulated. Numerous genes with increased transcript levels in heterozygous MEFs were identified to be involved in p53-dependent pathways. Furthermore, examination of the promoter regions of all up- and down-regulated genes revealed that the majority contained putative p53 responsive elements.

An analysis of transcript levels in MEFs after exposure to either ionizing or ultraviolet radiation revealed a significant change between wild type and heterozygous MEFS for Plk4 transcript levels upon only UV exposure. Furthermore, changes in protein levels of several important cell check-point and apoptosis regulators were examined, including p53, Chk1, Chk2, Cdc25C and p21. In heterozygous MEFs, p53, p21 and Chk2 protein levels were at significantly higher levels. Furthermore, p53 activity was increased 5 fold in the *Plk4 *heterozygous MEFs.

**Conclusion:**

Global transcript profiles and levels of key proteins involved in cellular proliferation and DNA damage pathways were examined in wild-type and *Plk4 *heterozygous MEFs. It was determined that Plk4 haploinsufficiency leads to changes in the levels of RNA accumulation for a number of key cellular genes as well as changes in protein levels for several important cell cycle/DNA damage proteins. We propose a model in which reduced Plk4 levels invoke an increase in p53 levels that leads to the aforementioned changes in global transcription profiles.

## Background

Plk4 (Sak), is a member of the polo-like kinase (Plk) family of serine/threonine kinases which are involved in the regulation of the cell cycle, cellular response to stress such as DNA damage, and the duplication and maturation of centrosomes [1-4]. Deregulation of the Plks by overexpression, depletion via epigenetic silencing or loss of heterozygosity (LOH) has implicated them in the development of centrosome abnormalities and has been associated with a CIN (chromosomal instability) phenotype and malignancy. Plk4 is a major regulator of centriole duplication as indicated first by an increase in the number of supernumerary centrosomes correlated with Plk4 overexpression, and second, by a reduction in centriole duplication with the eventual development of mono-polar spindles upon repeated cell divisions observed after RNA interference for Plk4 [5-8]. Homozygous null *Plk4 *mice are embryonic lethal at ~E7.5 of development, with an increase in the proportion of mitotic cells, whereas *Plk4 *heterozygous mice are phenotypically normal [9]. Interestingly aged *Plk4 *heterozygous mice display haploinsufficiency with tumours developing at a high frequency in major sites such as the liver and lung [10]. Haploinsufficiency for Plk4 affects normal progression through the cell cycle and maintenance of the genome. For example, in a two thirds liver hepatectomy model, *Plk4 *heterozygous hepatocytes had an increased rate of tri- and tetra-polar spindle complexes with frequent mitotic errors as compared to those form wild-type regenerating livers [10]. At 9–12 months post-hepatectomy all the *Plk4 *heterozygous mice had abnormal liver morphology and there was an increased rate of tumourigenesis [10]. These results suggest that Plk4 haploinsufficiency potentially leads to increased aneuploidy a likely tumour promoting event. Plk4 loss also has implications in human malignancy, where LOH for Plk4 was found in the majority of a small sample of hepatocellular carcinomas [10].

Plks 1–3 in general all play important roles in the regulation of the cell cycle and the DNA damage response. Furthermore, several of their respective substrates are in common, with the individual Plks likely placing their substrate under tighter or opposing control. For example, both Plk3 and Plk1 phosphorylate Cdc25C and p53 by targeting different residues in each case. Plk3 phosphorylates Cdc25C on serine 216 [11], a site that is also targeted by Chk1 and Chk2 [4,12]. Phosphorylation of serine 216 of Cdc25C is inhibitory, which is due to sequestration of the protein phosphatase in the cytoplasm by 14-3-3 protein, thus blocking mitotic entry [13]. Human Cdc25C is phosphorylated on Ser-198 by Plk1, part of an activation amplification loop that increases the phosphatases activity to allow mitotic entry [14]. Polo-like kinase 1 (Plk1) is known to inhibit p53 function by physical interaction [15], while phosphorylation of p53 at Ser 20 by Plk3 serves to functionally link DNA damage with increased p53 activity [16]. Chk2 is another protein that is phosphorylated by the Plks. Plk1 interacts with, phosphorylates and colocalizes with Chk1 [17], Plk3 phosphorylates Chk2 at two residues, which results in subsequent phosphorylation of Chk2 on T68 by ATM in response to DNA damage, thus upregulating Chk2 activity [18,19].

Similar to the other Plk family members, which have established roles in DNA damage pathways, Plk4 likely functions within or is a target of DNA damage pathways. This is supported by the observation that Plk4 interacts with and phosphorylates p53 [10,20]. Plk4 expression is repressed in a p53 dependent manner in response to DNA damaging agents, with the p53 repression of Plk4 activity occurring through the recruitment of a histone deacetylase (HDAC) transcription repressor [21]. Additionally, Cdc25C, a key regulator of the entrance into mitosis and target of DNA damage proteins, is a substrate for Plk4 [22]. Significant phenotypic differences are also observed between *Plk4 *wild-type and heterozygous mouse embryonic fibroblasts (MEFs) [10]. Contrary to what would be expected, heterozygous *Plk4 *MEFs display a phenotype typified by multiple centrosomes which lead to multipolar spindles, mitotic failure and delayed proliferation [10].

All the evidence published to date is consistent with a model as suggested by Habendanck et al (2005) in which reduced Plk4 activity causes occasional cellular division failure as a result of aberrant centrosome duplication and subsequent mitotic spindle malformation[7], This cell division failure can lead to either aneuploidy or polyploidy, which could in turn contribute to the higher incidence of tumors in heterozygous mice. As an initial step in further characterizing the effect of lower Plk4 levels on the cell, we utilized microarrays to provide a general survey of differences in the transcript profiles of *Plk4 *wild-type and heterozygous MEFs. Here, we report on a spectrum of genes that are upregulated or downregulated in the *Plk4 *heterozygous MEFs, including the key cell cycle regulators p53, p21 and chk2 and the presence of increased p53 levels/activity as a result of Plk4 haploinsufficiency.

## Methods

### Establishment of primary mouse embryonic fibroblasts (MEFs)

Mouse embryonic fibroblast cell lines were established from 12.5 day old wild-type and heterozygous Plk4 embryos as previously described [9]. All experiments utilizing mice as well as embryos and cell lines derived from them were performed in accordance to CCAC guidelines and approved by the University of Windsor Animal Care Committee. The MEFs were cultured in Dulbecco's Modified Eagles Medium (Sigma) containing 20% fetal bovine serum (Sigma), 1% penicillin-streptomycin (Gibco) and 250 ug/ml gentamicin (Gibco) and maintained at 37°C with 5% CO_2_. All experiments were performed with MEFs at passage 2–3.

### Flow cytometry

Wild type and heterozygous mouse embryonic fibroblasts were grown to approximately 80% confluency. The MEFs were then harvested, fixed in 80% ice-cold ethanol, stained with PI and the cell cycle profiles were determined by flow cytometry on a Beckman Coulter Cytomics FC 500 flow cytometer. Flow cytometry results were analyzed using Cytomics RXP Analysis software (Beckman Coulter). Presented results are based on three independent experiments.

### Microarray analysis

MEF cells were grown asynchronously to a confluency of 70–80% with total RNA isolation performed using the RNeasy Mini Kit (Qiagen). In order to confirm the integrity and quality of the RNA, the RNA was run on the 2100 Bioanalyzer (Agilent) using the RNA 6000 Nano Assay Kit. Total RNA extracted from MEFs was subjected to microarray analysis at the University Health Network (UHN) Microarray Centre in Toronto. The samples were labeled using the UHN's standard indirect labeling protocol and hybridized to a Mouse 22.4K chip. Results are based on three independent replicates with subsequent analysis performed using "The Institute for Genomic Research (TIGR) microarray software suite".

### Semi-quantitative RT-PCR

Reverse transcription (RT) was performed using Superscript II Reverse Transcriptase (Invitrogen) as per the manufacturer's instructions. All forward and reverse primers for PCR were designed to span intron/exon boundaries in order to prevent amplification of contaminating genomic DNA in the cDNA mixture. Primers for the amplification of Plk4, glyceraldehyde 3-phosphate dehydrogenase (GAPDH), Prohibitin, Sap30 Binding Protein (SAP30BP), and Wnt-Inducible Signaling Pathway Protein (WISP1) and the size of their respective products are summarized in Table 1. PCR was performed using Hot Start Taq DNA Polymerase (Qiagen) and the results of each amplification were normalized to the GAPDH internal control.

**Table 1:** Oligonucleotide primer sequences for RT-PCR analysis.

**Gene**	**Primers**	**Size (bp)**
**GAPDH**	Forward 5'GCTGAGTATGTCGTGGAGTCT-3' Reverse 5'-CAGAGCTGAACGGGAAGCTC-3'	**410**
		
**Plk4**	Forward 5'-AGGGAAGCTAGGCACTTCATG-3' Reverse 5'-GGAAGACCACCTTTTGAC-3'	**310**
		
**Sap30**	Forward 5'-CCAGAAGCTCTACGAGCGGAA-3' Reverse 5'TGGTCTGAAGACTCCTACTATGAG-3'	**190**
		
**Prohibitin**	Forward 5'-CGTATCTACACCAGCATTGGC-3' Reverse 5'-TGTGGTGGAAAAGGCTGAGC-3'	**301**
		
**Wisp1**	Forward 5'-GCCTAATCACAGATGGCTGTG-3; Reverse 5'-CAATAGGAGTGTGTGCACAGGTG-3'	**150**

### Exposure of MEFs to DNA damaging agents

Wild-type and heterozygous MEFs were exposed to ultraviolet light (UV) at 40 mJ/cm^2 ^using a GS Gene Linker UV Chamber (Biorad) or ionizing radiation (IR) of 25 Gy using a RX-650 Cabinet X-ray System (Faxitron) and RNA or protein was isolated from the MEFs at the specified time points.

### Western blot analysis

Cells were lysed in lysis buffer (50 mM Tris-Cl, 100 mM Nacl, 500 mM EDTA, 1% Triton-X), the cell lysate was cleared by centrifugation and equal amount of total protein was loaded into 8% (or 12%) SDS-PAGE gels. Following separation, proteins were transferred onto a PVDF membrane, and Western blot analysis was performed using standard methods. The primary antibodies were as follows, anti-p53 (Sigma), anti-Chk2 (Sigma), anti-Chk1, (Sigma), anti-p21 (BD Pharmingen), anti-Cdc25C (Santa Cruz) and anti-GAPDH (Cell Signalling). The secondary antibodies were as follow: anti-mouse HRP (Amersham), anti-rabbit HRP (Amersham) and were used at dilutions recommended by manufacturers.

### Apoptosis assay

The level of apoptosis was determined in heterozygous and wild-type Plk4 MEFs using a TdT-mediated dUTP Nick-End Labeling (TUNEL) assay as per the manufacturer's provided protocol (Promega). Cells were exposed to 40 mJ/cm^2 ^to induce UV mediated DNA damage and analyzed 1 hr, 2 hr, 4 hr, 6 hr, and 8 hr post radiation. Results are based on three independent experiments.

### X-gal senescence staining

MEFs were grown to 70–80% confluency and then stained using a β-Galactosidase Staining Kit (Cell Signalling). Cells were washed in PBS, fixed in a 2% formaldehyde solution and incubated overnight in 20 mg/ml X-gal staining solution. Senescent cells were identified by the presence of a typical perinuclear blue stain.

### p53 activity assay

The activity of p53 from *Plk4 *heterozygous and wild-type MEFs was analyzed with the Active p53 Activity Assay Kit (R*#38;D Systems). Cells were grown to 70–80% confluency and 5 ug of nuclear extracts (equal amounts of protein were determined by Bradford assay, Biorad) were subjected to the capture ELISA assay as per the manufacturer's protocol. Absorbance measurements were performed at 450 nm on a Victor 1420 Spectrophotometer. Results are based on three independent experiments and normalized to the wild-type controls.

## Results and discussion

### Comparison between transcript profiles in wild-type and heterozygous *Plk4 *MEFs using microarray

**Figure 1:**
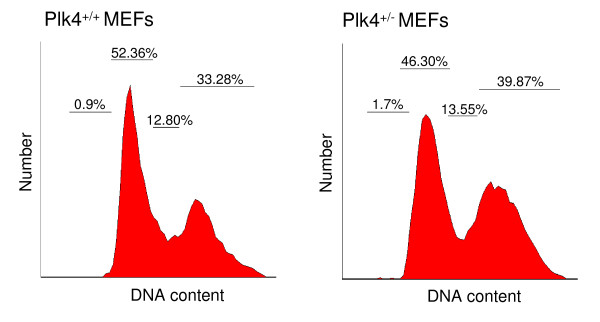
**Cell cycle profile of *Plk4 *heterozygous and wild-type MEFs**. Presented are typical cell cyle profiles of asynchronous populations of *Plk4 *heterozygous and wild-type MEFs based on flow cytometry of propidium iodide stained cells. The data is representative of three independent experiments.

A number of phenotypic differences have been observed between wild-type and heterozygous *Plk4 *MEFs [10]. For example, heterozygous MEFs exhibit a growth rate approaching one half that of their wild-type counterparts [10]. Many of these cells contain multiple centrosomes, micronuclei and mitotic defects [10]. In the present study we examined the cell cycle profiles of *Plk4 *heterozygous MEFs in comparison to wild-type MEFs and found that the heterozygous MEFS displayed a decrease in cells in G0/G1 and an increase in the number of cells in G2/M (Figure 1). These results suggest that Plk4 haploinsufficiency may also lead to impaired progression through the cell cycle with the potential to lead to abnormal chromosomal alignment and segregation. Plk4 haploinsufficiency and loss of heterozygosity have also been implicated in the development of primary hepatocellular carcinoma in mice and humans respectively [10].

The main focus of the present study was to examine global changes in transcript profiles between *Plk4 *wild type and heterozygous MEFs. In order to accomplish this, we utilized independent cultures of asynchronously growing age matched *Plk4 *wild-type and heterozygous MEFs in three replicates. Quantification and normalization of the data was performed using "The Institute for Genomic Research" (TIGR) TM4 microarray data analysis suite. Normalization and filtering of the data was performed using the TIGR Microarray Data Analysis System (MIDAS) application. Analysis of all microarray data sets for the different microarray experiments (ex. Wild-type *Plk4 *MEFs vs Heterozygous *Plk4 *MEFs) were performed independently.

K-means clustering analysis was performed using TIGR Multiexperiment Viewer (MEV). Within each cluster, genes having a log ratio value greater than 1 or less than -1 on each microarray chip were identified. Genes having a log ratio greater than 1 represented genes in the heterozygous MEFs that have at least a two fold increase in gene expression. As the wild-type MEFs was used as the control, genes with a log ratio greater than 1 were classified as up-regulated in the heterozygous MEFs and genes with a log ratio less -1 represented genes in the wild-type MEFs that have at least a two fold increase in gene expression or are down-regulated in the heterozygous MEFs.

**Figure 2:**
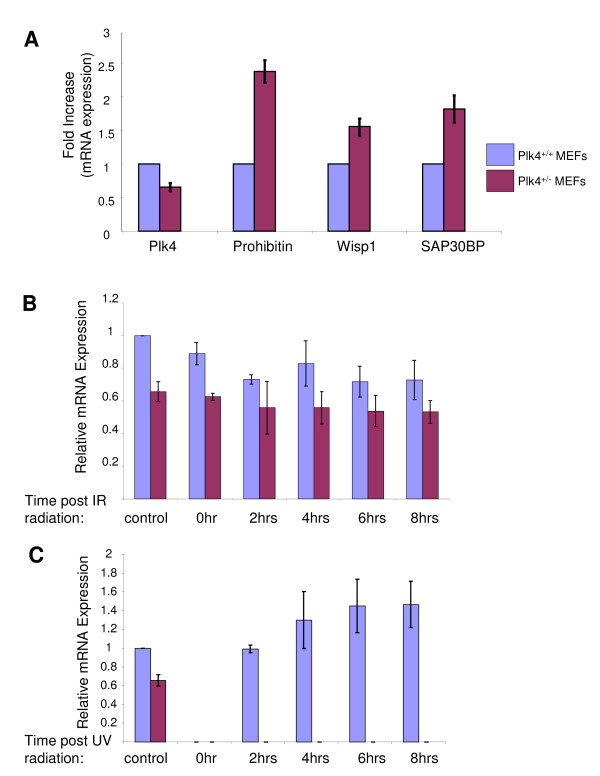
**Comparison of transcript levels between *Plk4 *heterozygous and wild-type MEFs**. **A) Confirmation of microarray results**. Semi-quantitative RT-PCR was carried out to measure the relative difference in the expression of Plk4 and the candidate genes prohibitin, wisp1 and sap30bp in *Plk4 *wild-type and heterozygous MEFs. Values in each case were normalized to the levels of GAPDH transcript. Presented data are a result of three independent experiments. **B) Relative levels of Plk4 transcript post ionizing radiation (IR)**. MEFs were exposed to 25 Gy IR and RNA was isolated at the indicated time points post exposure followed by RT-PCR to measure the relative abundance of Plk4 transcript (as above). **C) Relative levels of Plk4 transcript post ultraviolet radiation (UV)**. MEFs were exposed to 40 J/Cm^2 ^UV and RNA was isolated at the indicated time points post exposure followed by RT-PCR to measure the relative abundance of Plk4 transcript (as above).

From the microarray data, 9 genes were identified as having at least a two-fold decrease in transcript levels in the heterozygous MEFs when compared to the wild-type control (Table 2), while 143 genes were identified as having at least a two-fold increase in transcript levels in the heterozygous MEFs (Table 2). We ordered these genes within each functional group based on the average fold difference between *Plk4 *wild-type and heterozygous MEFs. In order to test the validity of the microarray results we assessed transcript levels for Plk4 and three arbitrary genes with at least a two-fold increase in expression by qualitative RT-PCR. A graphic representation of these results is shown in Figure 2A. In confirmation of previously published results [10], Plk4 transcripts levels in the heterozygous *Plk4 *MEFs are at about 60% of the level seen in wild-type MEFs. Furthermore, Prohibitin, Wisp1 and Sap30 are upregulated in the *Plk4 *heterozygous MEFs thus independently confirming their altered expression profile as identified by microarray results.

**Table 2:** Transcripts up and down regulated in Plk4 heterozygous MEFs.

**Upregulated Genes**							
**Cell Cycle**	**Gene ID**	**Fold Change**	**Putative p53 site**	**Metabolism**	**Gene ID**	**Fold Change**	**Putative p53 site**
Casein kinase II (Csnk2a1)	12995	5.8040	20	N-acylsphingosine amidohydrolase (acid ceramidase) like (Asahl)	11886	6.0539	14
Protein phosphatase 1F (PP2C domain containing) (Ppm1f)	68606	4.8644	10	Leucyl/cystinyl aminopeptidase (Lnpep)	240028	4.8539	6
Squamous cell carcinoma antigen recognized by T-cells 1 (Sart1)	20227	4.6888	20	Galactose-4-epimerase (Gale)	74246	4.7700	8
Origin recognition complex, subunit 4-like (Orc4l)	26428	4.5661	9	L-2-hydroxyglutarate dehydrogenase (L2hgdh)	217666	4.6257	15
Inhibitor of DNA binding 2 (Id2)	15902	4.4323	11	Fatty acid desaturase 3 (Fads3)	286922	4.2976	27
Protein phosphatase 5 (Ppp5c)	65179	4.0765	15	Carbohydrate sulfotransferase 2 (Chst2)	54371	4.2926	18
heme binding protein 2 (Hebp2)	56016	3.8955	10	Stearoyl-Coenzyme A desaturase 1 (Ankrd13c)	433667	3.9806	16
Neuropilin (Nrp1)	18186	3.0956	13	CCR4 carbon catabolite repression like 4 (Ccrn4l)	310395	3.7939	14
Prohibitin (Phb)	18673	3.0459	18	protein kinase, cAMP dependent regulatory, type I beta (Prkar1b)	19085	3.3199	18
Cyclin dependent kinase 8 (Cdk8)	264064	3.0094	17	**DNA Repair**			
Heterogeneous nuclear ribonucleoprotein C (Hnrpc)	15381	2.5962	8	Uracil-DNA glycosylase (Ung)	22256	4.1846	15
TVMSFG fibroblast growth factor receptor 1 precursor (Fgfr1)	14182	2.2643	18	MutS homolog 6 (Msh6)	17688	2.1811	23
Phosphatidylinositol 3-kinase (Pic3c2a)	18704	2.1636	9	Thymine DNA glycosylase (Tdg)	21665	2.0156	10
Pituitary tumor-transforming 1 (Pttg1)	30939	1.9518	11				
**Development**				**Transcriptional/Translational Regulation**			
Sal-like 3 (Sall3)	20689	7.9335	13	Cysteinyl-tRNA synthetase (Cars)	27267	4.8072	18
T-cell factor 4 (Rab27b)	80718	4.7804	14	Zinc Finger Protein 451 (Zfp451)	98403	4.1537	9
Nuclear receptor co-repressor 1 (Ncor1)	20185	4.4658	16	Tetratricopeptide repeat domain 1 (Ttc1)	66827	4.0003	20
Inositol 1,4,5-triphosphate receptor 5 (Itpr2)	16439	3.6909	10	Highly similar to CBP_MOUSE CREB-binding protein (Crebbp)	12914	3.7483	9
Procollagen, type VI, alpha 3 (Col6a3)	12835	3.5828	15	Zinc finger protein 689 (Zfp689)	71131	3.4636	17
Fetal Alzheimer antigen (Bptf)	207165	3.3594	NA	Glutamyl-prolyl-tRNA synthetase (Eprs)	107508	3.3065	19
WNT1 inducible signaling pathway protein 1 (Wisp1)	22402	3.2886	22	GC-rich sequence DNA-binding factor homolog isoform 1 (C21orf66)	67367	3.0984	N/A
T-box transcription factor Tbx15 (Tbx15)	21384	2.9248	13	Phenylalanine-tRNA synthetase 2 (Fars2)	69955	3.0678	13
Nuclear factor I/X (Nfix)	18032	2.8883	9	GLIS family zinc finger 3 (Glis3)	226075	2.6233	10
Thrombospondin 2 (Thbs2)	21826	2.5437	23	Transmembrane and tetratricopeptide repeat containing 2 (Tmtc2)	278279	2.2096	18
Osteopontin (Spp1)	20750	2.4717	15	Transcription factor A (Tfam)	21780	2.0680	14
Fukuyama type congenital muscular dystrophy homolog (Fktn)	246179	2.2362	9				
**DNA Methylation**				**Cellular/Ion Transport**			
SAP30 binding protein (Sap30bp)	57230	2.9834	8	Pleckstrin (Plek)	56193	6.0575	11
SET domain ERG-associated histone methyltransferase (Olfml3)	99543	2.0889	9	Syntaxin 18 (Stx18)	53407	5.0970	18
Calcium binding and coiled coil domain 1 (Calcoco1)	67488	5.6489	19	Aquaporin-1 (Aqp1)	11826	4.4128	16
				Solute carrier family 6 (Slc6a6)	21366	4.4110	18
**Miscellaneous Cellular Functions**				Exocyst complex component 3 (Exoc3)	211446	3.9296	10
Coiled Coil domain containing 131 (Ccdc131)	216345	6.4372	11	Protein-coupled receptor 19 (Gpr19)	14760	3.8281	21
Thyroid hormone receptor interactor 11 (Trip11)	109181	5.8691	NA	Solute carrier family 39 (Slc39a10)	227059	3.5028	13
Smg-6 homolog (Smg6)	103677	5.3375	11	Frequenin homolog (Freq)	14299	3.1881	15
Talin 2 (Tln2)	70549	5.2780	N/A	Serine Hydrolase like (Serhl)	68607	3.1718	17
Tomoregulin 1 (Tmeff1)	230157	5.1116	7	Solute carrier family 14 (Slc14a2)	27411	3.0842	14
Channel-interacting PDZ domain protein (Inadl)	12695	4.9504	14	Translocator of inner mitochondrial membrane (Timm17b)	21855	2.7129	19
WD repeat domain 50 (Utp18)	217109	4.9252	16	Similar to crooked neck protein (Ipo7)	233726	2.5873	17
Inositol hexaphosphate kinase 1 (Ip6k1)	27399	4.8086	12	Oxysterol binding protein like protein 9 (Osbpl9)	100273	2.5501	12
Spetex-2E protein (100040875)		4.6574	N/A	ATPase, Ca++ transporting, plasma membrane 2 (Atp2b2)	11941	2.4328	26
Multiple PDZ domain protein (Mpdz)	17475	4.0596	12	Transient receptor potential cation channel, subfamily M, member 7 (Trpm7) 58800		1.9917	11
Myosin heavy chain 10 (Myh10)	77579	3.8880	11				
CDC42 effector protein (Rho GTPase binding) 2 (Cdc42ep2)	104252	3.5581	23	**Downregulated Genes**			
Zinc finger protein 507 (Zfp507)	668501	3.4688	N/A	**Development**			
aarF domain containing kinase 1 (Adck1)	72113	3.4641	9	Procollagen, type III, alpha 1 (Col3a1)	12825	-4.6364	6
AHNAK nucleoprotein (Ahnak)	66395	3.2688	20	Procollagen, type V, alpha 2 (Col5a2)	12832	-4.1420	12
Ring finger protein 11 (Arhgdia)	192662	3.1199	25	Oral-facial-digital syndrome 1 gene homolog (Ofd1)	237222	-3.0782	8
Villin (Vil1)	22349	2.9808	15	Procollagen, type I, alpha 2 (Col1a2)	12843	-2.9648	12
3-phosphoglycerate dehydrogenase (Phgdh)	236539	2.9427	18	**Metabolism**			
Arginine/serine-rich coiled-coil 1 (Rsrc1)	66880	2.9404	6	Stearoyl-Coenzyme A desaturase 2 (Scd2)	20250	-2.4729	10
Olfactory receptor 202 (Olfr202)	258997	2.9278	7	Mus musculus mVL30-1 retroelement mRNA sequence (mVL30-1)		-2.5566	N/A
Discs, large homolog 5 (Dlg5)	71228	2.9094	N/A	Transmembrane protein 34 (Tmem184c)	234463	-2.5241	14
2'-phosphodiesterase (E430028B21Rik)		2.8426	0	Mus musculus 0 day neonate cerebellum cDNA (E430024C06Rik)	319443	-2.3000	N/A
HD domain containing 3 (Hdcc3)	68695	2.7345	14	Hypothetical protein LOC639390 (LOC639390)		-2.2121	N/A
Heat shock protein 1(Heatr1)	217995	2.7243	13				
Myotubularin related protein 7 (Mtmr7)	54384	2.4370	8				
Mitochondrial ribosomal protein L50 (Mrpl50)	362517	2.2540	20				
Proteasome(macropain)26S subunit, non-ATPase (Psmd4)	19185	2.2031	11				
NICE-5 protein (AA414768)	245350	2.1272	N/A				

Shown above are ninety-seven upregulated genes and nine downregulated genes in the heterozygous MEFs. "Fold change" is the change in transcript level in the heterozygous MEFs relative to wild-type MEFs as determined by microarray analysis. The "Putative p53 sites" are as determined by MAPPER and is based on the first 5000 nucleotides upstream of the transcriptional start site. Please note that forty-six downregulated transcripts and their corresponding gene were omitted from the table as they encode hypothetical proteins or have unknown cellular functions. All the microarray data has been deposited into the MIAME database under accession number E-MEXP-2161.

The presumed major cellular function for each down or up-regulated gene was identified using annotation data from the PubMed database, and/or the Online Mendelian Inheritance in Man (OMIM) database. Furthermore, we utilized GenMAPP 2 [23] and Panther [24] to identify global biological trends in our gene expression data. The few genes that were downregulated in *Plk4 *heterozygous MEFs functionally included genes involved in development and metabolism. Far more genes were upregulated than were downregulated in the heterozygous MEFs. These included genes with a spectrum of known functions such as cell cycle control, the DNA damage response, DNA repair, epigenetic modification, development, and transcription/translation. In particular, several key genes involved in p53 dependent pathways, Rho signaling, Wnt signaling and the proteasome were upregulated in the heterozygous MEFs. Several of these genes have been implicated in malignancy and are of particular interest given the increased rate of malignancy previously identified in *Plk4 *heterozygous mice. This includes securin (*Pttg1*) which serves to prevent premature chromosome separation through inhibition of separase activity. Securin is involved in several key cellular events including mitosis, cell cycle progression, DNA repair and apoptosis. Furthermore, securin (*Pttg1*) is upregulated in several malignancies and in particularly, pituitary adenomas [25]. Casein Kinase II (Csnk2a1), a serine/threonine kinase is a positive regulator of Wnt signalling pathway that is also upregulated in most cancers [26]. Phosphatidylinositol 3-kinase (Pic3c2a) is an upstream regulator of Akt [27], both of which are aberrantly regulated in many cancer types and as such are prime targets for intervention [28], Wisp1 overexpression has been implicated in cellular morphological transformation [29] and hepatocellular carcinoma [30].

The observation that the expression levels of genes involved in p53 dependent pathways were altered, coupled with the known interaction of p53 with Plk4, and since changes in p53 levels, like Plk4, may also contribute to centrosome abnormalities [24,31,32], led us to further analyze this result. We therefore analyzed the promoter region of both the up and downregulated genes utilizing the MAPPER search engine [33,34] and found that the majority of these genes contained numerous p53 responsive elements within the first 5 kilobases upstream of the transcriptional start site. Furthermore, several of these upregulated genes are known p53 targets (including *msh2 *[35]) or affect the p53 transcriptional machinery (like CDK8 [36]).

### The effect of ionizing and ultraviolet radiation on the Plk4 transcript and protein profiles in MEFs

**Figure 3:**
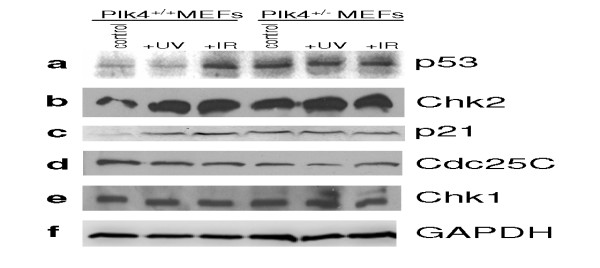
**Comparison of protein levels upon DNA damage between heterozygous and wild-type MEFs**. Heterozygous and wild-type *Plk4 *MEFs were exposed to 25 Gy IR or 40 mJ/cm^2 ^UV. Six hours post exposure cell extracts were subjected to Western Blot analysis. Shown are representative data from 3 repeats. GAPDH was used as a loading control to ensure equal protein loading.

The Plks are known components of DNA damage pathways, affecting levels and activity of a number of key proteins including p53, Chk2, Cdc25C and others. Plk4 is known to interact with proteins involved in the response to DNA damage including p53 [20], Cdc25C [22] and Chk2 [37]. This characteristic, coupled with the observation that many of the upregulated genes contain numerous p53 responsive elements within their promoter, led us to examine the effect of DNA damaging agents on the levels of key genes involved in the DNA damage response in the context of Plk4 levels. Wild-type and heterozygous MEFs were exposed to 25 Gy IR or 40 mJ/cm^2 ^UV and changes in transcript levels were analyzed by semi-quantitative RT-PCR. In response to IR, we found no significant difference in Plk4 transcript levels between wild-type and heterozygous MEFs (Figure 2B). In contrast, upon exposure to UV, there is a striking difference in transcript profile levels of Plk4 between *Plk4 *wild-type and heterozygous MEFs (Figure 2C). Initially, post exposure to UV, Plk4 transcripts are undetectable in both wild-type and heterozygous MEFs. Interestingly two hours post UV exposure plk4 transcripts levels in the wild-type MEFs returned to control levels and then subsequently increase to 40% greater expression, while no Plk4 transcripts are detectable in the heterozygous MEFs. Unfortunately, we were unable to detect Plk4 protein in these MEFs with the commercially available antibodies. As our array results suggest that p53 activity may be increased in the *Plk4 *heterozygotes, we were therefore interested in examining p53 protein levels. We found a sharp contrast between wild-type and heterozygous MEFs, with levels of p53 protein expression substantially higher in the heterozygotes (Figure 3a). These results would seem to suggest that the levels of p53 in the heterozygous MEFs may be the result of DNA damage that occurs as a result of genomic instability. We also examined p53 levels in these cells after exposure to DNA damaging agents based on the differences found for Plk4 transcript levels 6 hrs post exposure. In the case of UV we did not see any additional increase in the level of p53 protein compared to the *Plk4 *wild-type and heterozygote controls. In contrast, the levels of p53 in the wild-type MEFs increased to the heterozygote levels when exposed to IR. The levels were stabilized at close to maximal levels for these conditions and increased insignificantly. Results displayed are the representative data from three independent experiments. We subsequently examined the levels of a number of known cell cycle control and DNA damage response proteins. Chk2, which is known to aid in the maintenance of sustained G_1_, G_2_/M arrest, and apoptosis by phosphorylating p53 [12,38-41], was also at much higher levels in the heterozygous MEFs pretreatment (Figure 3b). Upon exposure to UV or ionizing radiation the levels of Chk2 were elevated in both wild-type and heterozygous MEFs. We next examined the levels of p21, a downstream effector and transcriptional target of p53 and found that the levels were increased in *Plk4 *heterozygote MEFs when compared to wild-type (Figure 3c). Consistent with this result, in our microarray data, p21 transcript levels were also upregulated 1.95 times (just below our arbitrarily chosen 2 fold cut-off) in the *Plk4 *heterozygous MEFs. Additionally, six hours post DNA damage we observed a substantial increase in p21 levels in the wild-type MEFs, while there was no further increase apparent in the heterozygous MEFs post DNA damage. The observation that p21 protein and transcript levels were elevated in the heterozygous MEFs again corresponds with the aforementioned increased levels of p53. Furthermore, it is also consistent with the increased levels of CDK8 transcripts found in the heterozygous MEFs (Table 2). CDK8 binding to p53 target genes is known to correlate positively with transcriptional strength. CDK8 is recruited to the *p21 *locus during conditions of strong p21 transcriptional activation [36]. We found minimal differences in the detectable levels of Cdc25C (Figure 3d) and Chk1 (Figure 3e) prior to or post treatment.

### The effect of UV induced DNA damage on apoptosis and cell cycle profiles in *Plk4 *MEFs

**Figure 4:**
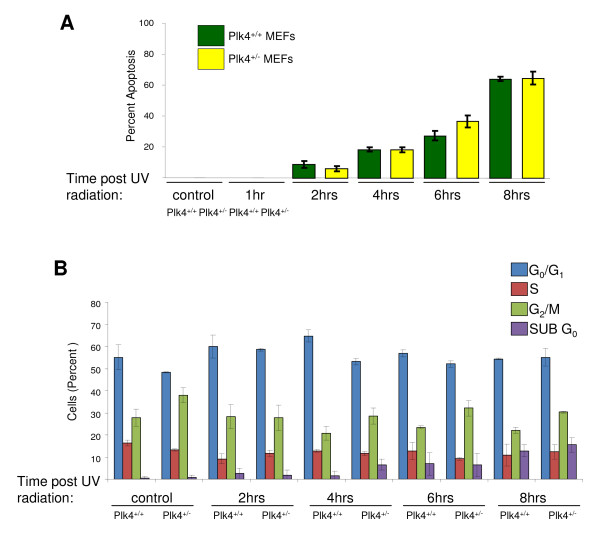
**Assessment of levels of apoptosis and cell cycle arrest in heterozygous and wild-type *Plk4 *MEFs**. **A.) Levels of apoptosis after UV induced DNA damage in *Plk4 *heterozygous and wild-type MEFs**. Apoptosis was analyzed prior to and after UV induced DNA damage (40 mJ/cm^2^) with a TUNEL assay. The results are presented as a percentage of apoptosis positive cells and are representative of three independent experiments. **B.) Effect of UV induced DNA damage in *Plk4 *heterozygous and wild-type MEFs on progression through the cell cycle**. Cell cycle profiles were analyzed prior to and after UV induced DNA damage (40 mJ/cm^2^) by flow cytometry after staining of the DNA with propidium iodide. The population of cells in G_0_/G_1_, S, G_2_/M and Sub G_0 _are displayed as percentage and are representative of three independent experiments.

As p53 is a major regulator of apoptosis we were next interested in determining whether the levels of apoptosis were higher in the *Plk4 *heterozygous MEFs. Since we observed a large decrease in the level of plk4 transcripts in heterozygous MEFs upon UV exposure and since UV is a more effective inducer of both p53 activity and apoptosis than IR [42] we examined the levels of apoptosis after UV treatment. Interestingly, we found no significant difference in the levels of apoptosis for wild-type and heterozygous MEFs prior to or post treatment with UV (Figure 4A). While we were surprised that there was no difference in the level of apoptosis between wild-type and heterozygous MEFs, it is consistent with the elevated level of Wisp1 mRNA seen in the heterozygous MEFs. Wisp1 is known to attenuate p53 mediated apoptosis through the activation of PKB/Akt anti-apoptotic pathways [43]. This results in the protection of cells from the late stages of p53 mediated apoptosis. Additionally, while the cell cycle profiles of wild-type and heterozygous MEFs were different without treatment, their respective responses to UV irradiation were similar with an overall increase in the Sub G0 population post exposure (Figure 4B).

### Senescence and p53 activity in Plk4 MEFs

In most cell types p53 is a potential key regulator of senescence growth arrest, the maintenance of senescence growth arrest and the initiation of the senescence response following DNA damage [44]. In order to address the possibility that elevated p53 levels may be correlated with a senescent phenotype in the *Plk4* heterozygous MEFs, we stained the cells for β-galactosidase activity from passages 2–5 (see additional file 1). We found no evidence of increased β-galactosidase activity in the *Plk4 *heterzoygous MEFs relative to the wild type MEFs; thus suggesting that the elevated p53 protein levels in the *Plk4 *heterozygous MEFs were not correlated with an increase in cellular senescence.

**Figure 5:**
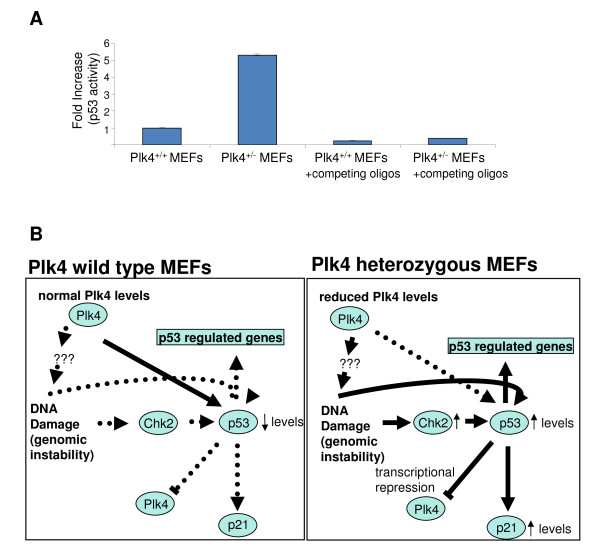
**p53 activity and model of Plk4 and p53 interactions in *Plk4 *heterozygous MEFs**. **A.) Effect of Plk4 haploinsufficiency on p53 activity**. p53 transcriptional activity was analyzed in Plk4 heterozygous and wild-type MEFs from nuclear extracts using a capture ELISA assay. The p53 specificity was confirmed upon incubation with competing labeled and unlabeled oligonucleotides. The results were normalized against wild-type control and are representative of three independent experiments. **B.) Model of p53 and Plk4 regulation in *Plk4 *heterozygous and wild-type MEFs**. Shown are proposed Plk4/p53 direct/indirect interactions in *Plk4 *wild-type and heterozygous MEFs with the resultant corresponding changes in protein levels, activity and gene expression. Solid lines indicate pathways that are functionally active, whereas dotted lines indicate pathways with reduced activity.

p53 accumulation and activity is regulated by post-translational modification of at least 20 sites via protein phosphorylation and/or other post-translational modifications [42]. As previously stated, the p53 protein levels are markedly increased in the *Plk4 *heterozygous MEFs compared to wild-type. It was therefore of interest to determine whether the increase in p53 levels was also accompanied by an increase in p53 activity. In order test this possibility we utilized an enzyme-linked immunosorbent assay (ELISA) to measure p53 transcriptional activity in our *Plk4 *wild-type and heterozygous MEFS. Interestingly, in agreement with the presence of p53 responsive elements in the genes that were upregulated in the array data, we observed a 5 fold increase in p53 activity in the heterozygous MEFs (Figure 5A).

We propose that the increased p53 protein levels and activity that occur as a result of Plk4 haploinsufficiency may in turn contribute to the over-expression of numerous genes containing p53 responsive elements within their promoters (Figure 5B). The function of these genes encompasses a spectrum of cellular activities including cell cycle control and the response to DNA damage. The results suggest that one function of Plk4 phosphorylation of p53 may be with respect to p53 protein stability and/or activity. In this scenario the possibility exists that this arises through a direct effect in which lower Plk4 levels result in reduced phosphorylation of p53 by Plk4 thus leading to an increase in protein p53 stability and activity. Alternatively, the presence of supernumerary centrosomes seen in *Plk4 *heterozygous MEFs may lead to an increase in genomic instability and the induction of checkpoints to deal with the ensuing DNA damage. Very few targets for Plk4 have been identified thus far. However, the possibility exists that the increased levels of p53 and phenotypic changes observed occur as a result of indirect consequences of Plk4 haploinsufficiency and targeting of other substrates. For example, one known plausible indirect effect of Plk4 haploinsufficiency could be through Chk2. Plk4 both interacts with and phosphorylates Chk2, a key regulator of the DNA damage response and p53 [37]. Conceivably, reduced phosphorylation of Chk2 as a result of lower Plk4 levels may result in altered Chk2 levels and/or activity towards p53 thus resulting in p53's increased stability or activity. This is consistent with the observation that Chk2 levels are greatly increased in the heterozygous MEFs and the observation that the cell cycle profiles of heterozygous MEFs are altered.

## Conclusion

In conclusion, our results demonstrate that Plk4 haploinsufficiency leads to changes in the levels of RNA accumulation for a number of key cellular genes as well as changes in protein levels for several important cell cycle/DNA damage proteins. The majority of the upregulated genes have numerous p53 responsive elements within their promoter regions, thus suggesting that Plk4 haploinsuficiency directly or indirectly leads to an increase in p53 activity in MEFs. Further studies should reveal the nature of the relationship between Plk4 levels, p53 and the down and upregulated genes found in *Plk4* heterozygous MEFs.

## Abbreviations

Cdc25C: Cell division cycle 25 homolog C; CDK8: Cyclin dependent kinase 8; Csnk2a1: Casein Kinase II; GAPDH: glyceraldehyde 3-phosphate dehydrogenase; HDAC: Histone deacetylase; LOH: Loss of heterozygosity; MEFs: Mouse embryonic fibroblasts; MEV: Multiexperiment viewer; MIDAS: Microarray Analysis System; OMIM: Online Mendelian Inheritance in Man; Pic3c2a: Phosphatidylinositol 3-kinase; Plk: Polo-like kinase; Plk4: Polo-like Kinase 4; RT-PCR: reverse trancription polymerase chain reaction; Sak-Snk/Plk-akin kinase; SAP30BP: Sap 30 binding protein; Sart1: Squamous cell carcinoma antigen recognized by T-cells; TIGR: The Institute for Genomic Research; TUNEL: TdT mediated dUTP Nick-End Labeling; UHN: University Health Network; UV: Ultraviolet; WISP1: Wnt Inducible Signaling Pathway protein.

## Authors' contributions

AM carried out the initial isolation of MEFs, along with the RNA isolation, RT-PCR, microarray data analysis and Western blot analysis of proteins of interest. AW isolated additional MEFs, performed the flow cytometry analysis, β-galactosidase assays, and the p53 activity assay. JN performed the analysis and determination of p53 responsive elements in all genes that were found to be up- or down-regulated in *Plk4 *heterozygous MEFs. JWH was involved in all aspects of the study including conception, design, and coordination of the study, along with drafting the manuscript. All authors were involved in reading and approving the final draft.

## Supplementary Material

Additional file 1 **Supplementary Figure 1**. Figure showing the results from the X-gal assay in plk4 wild type and heterozygous MEFs. Differences in senescence were determined at passages 3–5 for heterozygous and wild-type *Plk4 *MEFs by β-galactosidase assay. Senescing Human foreskin fibroblast (HFF-1) (passage 52) were used as a positive control. Arrows point to the characteristic blue perinuclear staining. Click here for file
