# Supplementary figures and images for: Gene expression patterns in heterozygous Plk4 murine embryonic fibroblasts
Source: BMC Genomics. 2009 Jul 16;10:319. doi: 10.1186/1471-2164-10-319 (PMC2727538; doi:10.1186/1471-2164-10-319)

## Slide 1
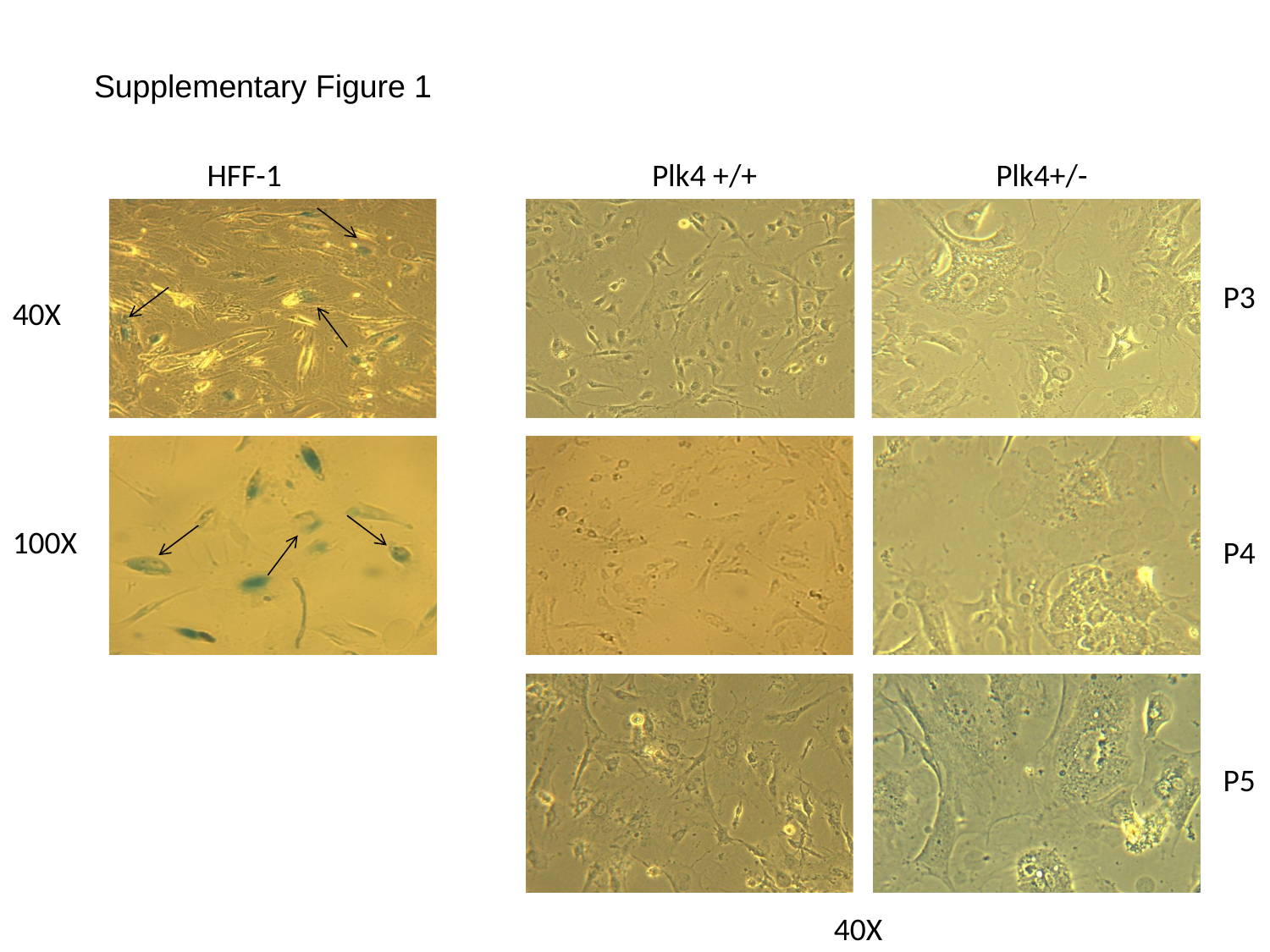

Supplementary Figure 1
HFF-1
Plk4 +/+
Plk4+/-
P3
P4
P5
40X
100X
40X

Supplement: Additional file 1 — Supplementary Figure 1. Figure showing the results from the X-gal assay in plk4 wild type and heterozygous MEFs. Differences in senescence were determined at passages 3–5 for heterozygous and wild-type Plk4 MEFs by β-galactosidase assay. Senescing Human foreskin fibroblast (HFF-1) (passage 52) were used as a positive control. Arrows point to the characteristic blue perinuclear staining. [file 1471-2164-10-319-S1.ppt]
